# A Peculiarly Interesting Case of Indigestion of the Stomach

**Published:** 1897-11

**Authors:** 


A PECULIARLY INTERESTING CASE OF INDIGESTION OF THE STOMACH (by Dr. Michelin).—This case was a pronounced one of indigestion of the stomach, and was caused by the animal drinking large quantities of water while very warm. A short time afterward the animal presented the following alarming symptoms: Entire loss of appetite; colic and nausea, followed by attempts at vomiting. The treatment consisted of alcoholic and camomilic injections, of ten centigrammes pilocarpine, and external frictions of terebene. In a short time the symptoms were much milder, but very soon returned with much greater force and producing at the same time profuse perspiration of the back and sides. The animal was given a drench containing acetate of ammonia, but the symptoms still continued acute and frequently violent. In half an hour large quantities of alimentary matter were ejected from the nose and had the peculiar odor of chyme. The author diagnosed rupture of the stomach, and expected the animal to die in a very short time. The next morning at five o'clock the animal walked twenty-five kilometres, and ate all of the food in the manger of the animal alongside of him, and in two days afterward was completely cured.

This combination rather conflicts with numerous other authors, showing that it is possible to have vomiting in the horse without rupture of the stomach.—*Recueil de Médecine Veterinaire.*

